# Rates and Predictors of Colorectal Cancer Screening

**Published:** 2006-09-15

**Authors:** Su-Ying Liang, Kathryn A Phillips, Mika Nagamine, Uri Ladabaum, Jennifer S Haas

**Affiliations:** School of Pharmacy, University of California, San Francisco; School of Pharmacy, University of California, San Francisco, San Francisco, Calif; Institute for Health Policy Studies, University of California, San Francisco, San Francisco, Calif; Division of Gastroenterology, University of California, San Francisco, San Francisco, Calif; Division of General Medicine and Primary Care, Brigham and Women’s Hospital and Harvard Medical School, Boston, Mass

## Abstract

**Introduction:**

Despite widespread recommendations for colorectal cancer screening, the U.S. screening rate is low. The objectives of this study were to describe the rates and predictors of colorectal cancer screening use by examining groups in two categories — 1) those who have ever been screened and 2) those with up-to-date screening — and to assess whether trends and predictors change over time.

**Methods:**

We analyzed data from the 2000 and 2003 National Health Interview Surveys about the use of fecal occult blood tests, sigmoidoscopies, and colonoscopies for adults aged 50 years and older and without a history of colorectal cancer (N = 11,574 in 2000 and N = 11,779 in 2003).

**Results:**

Rates in the 2000 study population of those who have ever been screened for colorectal cancer (53%) had increased in the 2003 study population (55%) as had the rates in the 2003 study population of those with up-to-date colorectal screening (53%) compared with the rates in the 2000 study population (38%). Among those who were ever screened, 76% were up-to-date with screening in 2003, compared with 68% in 2000. There was increased use of colonoscopies but decreased use of fecal occult blood tests and sigmoidoscopies. Individuals were more likely to be up-to-date with screening if they had higher income, higher education, insurance coverage, a usual source of care, and a dental visit in the last year than if these predictors were not evident. Since 2000, these predictors of colorectal cancer screening use have remained stable.

**Conclusion:**

Although there has been relatively limited success in increasing overall screening, it is encouraging that most people in the group of those who have ever been screened are up-to-date with colorectal cancer screening. Predictors for colorectal screening were stable over time despite changes in screening policies and rates. Further research is needed to uncover barriers to colorectal cancer screening.

## Introduction

Although colorectal cancer (CRC) is a common cancer in both men and women in the U.S. and although screening for CRC reduces mortality and is recommended by several professional organizations (e.g., American College of Gastroenterology, American Cancer Society) (1-5), the screening rate for CRC is low compared with screening rates for other cancers, such as breast and cervical (6-10). Measuring CRC screening rates is complex because multiple tests are used, and recommended intervals between tests vary. Studies that have examined predictors of CRC screening use have included individual characteristics (e.g., sociodemographic, behavior, risk factors) and health care system elements (e.g., having medical insurance, having a physician recommendation, having a usual source of care) (11,12). However, relatively little is known about whether the rates and predictors of CRC screening change over time. Understanding current screening rates and predictors is critically important because of recent changes in CRC screening policies. For example, an increasing number of state mandates require private insurers to cover CRC screenings (13), and the 2000 enactment of Medicare coverage for CRC screening (14) enables screening for adults aged 65 years and older.

The objectives of this study were to examine rates and predictors of CRC screening use by focusing on people who have ever been screened and those who are up-to-date with screening and to assess whether use rates and predictors changed from 2000 to 2003. We recognized that frequency of use implies test availability and availability of physicians with required training and specialty for invasive tests (e.g., sigmoidoscopies, colonoscopies). This study updates national estimates of screening rates (6,9) and predictors of CRC screening by using nationally representative data from the 2000 and 2003 National Health Interview Surveys (NHIS) (15,16). This study contrasts with previous studies of CRC screening in the following ways: 1) it focuses on two groups (those who have ever been screened and those with up-to-date screening) and examines differences in CRC screening behavior among these two groups; 2) it uses variables to examine CRC screening behavior and includes whether tests were used alone or in combination; 3) it assesses and statistically tests whether CRC screening use and predictors changed from one period of time to the next by examining data from 2000 and 2003. Other studies examine screening trends (9,13), but to our knowledge, this is the first study to empirically test whether there were differences in rates and predictors of CRC screening use between 2 years.

## Methods

### Data and sample

Analysis for this study was based on data from the 2000 and 2003 NHIS, which are nationally representative in-person household surveys of noninstitutionalized civilians conducted by the National Center for Health Statistics, Centers for Disease Control and Prevention (15,16). The NHIS collects information on household composition, income, assets, sociodemographic characteristics, health status and activity limitations, health care access and use, insurance, health behaviors, and other variables.

The NHIS Cancer Control Supplement, cosponsored by the National Cancer Institute (15), collects information on cancer behavior risk factors and cancer screening. A complete Cancer Control Supplement survey was administered in 2000. In 2003, selected cancer screening questions, including those about CRC, were repeated. The 2000 NHIS included 100,618 adults and children, and the household response rate was 88.9%. The 2003 NHIS included 92,148 adults and children, and the response rate was 89.2%. For purposes of this study, we analyzed adults aged 50 years and older without a history of colorectal cancer. The 2000 sample for this study included 11,574 individuals, and the 2003 sample included 11,779 individuals.

### Outcome variables

NHIS respondents aged 40 years and older were asked if they had ever had 1) a home fecal occult blood test (FOBT)NHIS respondents aged 40 years and older were asked if they had ever had 1) a home fecal occult blood test (FOBT) and 2) a colorectal examination (sigmoidoscopy, colonoscopy, or proctoscopy). If respondents replied that they had ever had any of the tests, they were asked to identify the name of the test or examination. Respondents were asked the month and year they had their last test. If test date information was unknown, a follow-up question was asked to identify the time frame of the most recent examination. Possible responses included the following: 1) 1 year ago or less, 2) 1 to 2 years, 3) 2 to 3 years, 4) 3 to 5 years, 5) 5 to 10 years, and 6) more than 10 years ago.

We focused on 1) people who were ever screened for CRC and 2) people who were up-to-date for CRC screening. The CRC tests included in our analysis were home FOBT, sigmoidoscopy, and colonoscopy performed for any reason on adults aged 50 and older. We specified adults aged 50 years and older as our study sample, and they were classified as the subpopulation in the survey estimation. Adults aged 40 to 49 were not included in our subpopulation because, while they may be at high risk for CRC, they were not the focus of this study. Double-contrast barium enema tests were not analyzed because those data are not collected in the NHIS. Proctoscopy was excluded because it is not currently recommended as a CRC test (4).

#### Individuals who have ever been screened

Respondents were classified as ever screened if the person had ever been screened by using any of the three tests (home FOBT, sigmoidoscopy, or colonoscopy). We used the following detailed, mutually exclusive categories based on tests performed alone or in combination: 1) FOBT only, 2) sigmoidoscopy only, 3) colonoscopy (with or without FOBT), 4) both FOBT and sigmoidoscopy, or 5) do not know or other test.

#### Individuals who are up-to-date with screening

Respondents were classified as being up-to-date with screening if they followed screening guidelines recommended by the American Cancer Society (4) by having a home FOBT test in the past 12 months, a sigmoidoscopy in the last 5 years, or a colonoscopy in the last 10 years. We analyzed patterns of up-to-date screening by using the following mutually exclusive groups: 1) those not screened within the recommended time frame, 2) those screened only by FOBT in the last 12 months, 3) those screened only by sigmoidoscopy in the last 5 years, 4) those screened by colonoscopy (with or without FOBT) in the last 10 years, and 5) those who had both FOBT and sigmoidoscopy (FOBT in the last 12 months and sigmoidoscopy in the last 5 years).

### Factors associated with screening use

The conceptual framework for the study was based on a behavioral model of health care use which is one of the most frequently used frameworks for analyzing factors associated with patient use of health care services. The model suggests that patients' use of health care services is a function of their predisposition to use services, factors that enable use of services, and their need for care (17,18). We hypothesize that CRC screening use is associated with individual-level variables that represent *predisposing*, *enabling*, and *need* factors.

The predisposing factors were sociodemographic characteristics, such as age (50–64, 65–74, ≥75), sex, race and ethnicity (Hispanic, white, African American, and Asian or other), household income (% of federal poverty level categorized into the following groups: low [less than 200%], medium [200%–399%], and high [400% or more]), education (less than high school graduate, high school graduate, college graduate or more), and an individual's use of preventive services (defined by had or did not have a dental check-up in the past year). The enabling factors were having health insurance coverage (private, Medicare, Medicaid or other public insurance, or uninsured) and having a usual source of care. Need factors included the following: 1) behavioral risk factors (alcohol consumption >720 drinks per year for men or >360 drinks per year for women, smoking, and lack of physical activity); 2) being overweight or obese (a body mass index [kg/m^2^] of 25 or more; 3) self-reported chronic conditions (diabetes, arthritis, ulcers, cardiovascular disease, hypertension, asthma or emphysema); and 4) self-reported health status (very good, good, fair, poor). We analyzed the categorical version of these factors in bivariate analysis. Selected variables were entered in the logistic regression in the form of continuous (age, education, number of behavioral risk factors, number of chronic conditions) or ordinal (household income) when appropriate for parsimony and efficiency.

### **Statistical **analyses

We hypothesized that these individual-level predisposing, enabling, and need variables would be associated with CRC screening use. We used χ^2^ tests and logistic regressions analyses to examine associations between individual characteristics and CRC screening use. Independent variables for regression models were chosen based on theory, statistical significance, and parsimony. We discuss results as statistically significant when *P* < .05. All regression analyses used sampling weights to reflect the U.S. civilian, noninstitutionalized population and standard error adjustment to account for the complex survey design. All calculations were performed using Stata version 8 (StatCorp, College Station, Tex).

We used the Chow test to investigate whether specific predictors have different effects across years (21-24). The advantage of the Chow test is that it allows statistical analysis to show whether coefficients are different rather than relying on estimates. The original Chow test uses an F test for detecting differences between coefficients in separate regressions (19). The F test evaluates all coefficients together and was initially designed for linear models.  In this study, we adopted an alternative approach that has been shown to be equivalent to the original Chow test and that allows flexibility to test whether individual factors have different effects across years (20-22). By using the 2000 and 2003 pooled NHIS data, we estimated models of screening use by adding a dummy variable for year and interactions between this dummy variable and other independent variables.


Equation (1) Y = α+β * χ+α' * D+β' * D*χ+u,

where Y = probability of screening for an individual or probability of being up-to-date with screening,

χ= a vector of independent variables,

D = a dummy variable indicating the year 2003,

D*χ = interaction terms between the dummy variable and independent variables,

α, β, α', β' = parameters characterizing this function to be estimated,

u = error term.

Testing whether coefficients in the 2000 model are equal to coefficients in the 2003 model is equivalent to testing coefficients of interaction terms being zero, β'= 0. As shown in equations 1a and 1b, coefficients of the interactions, β', represent the differences between the 2000 and 2003 models. For the purpose of testing whether coefficients changed between 2000 and 2003, the models were fitted using a linear probability model to avoid complexity of interpreting the interaction terms in nonlinear models (23).

Equation (1a), Year 2000, D = 0: Y = α+β * χ+u

Equation (1b), Year 2003, D =1: Y = (α+α') + (β+β') * χ+u

## Results

### Rates of screening use in 2000 and 2003

We observed increasing rates of people having ever been screened and people having up-to-date screening from 2000 to 2003 (Table 1). In 2003, the ever screened rate was 55%, up from 53% in 2000 (*P* = .10). Among the total study population, 42% were up-to-date with screening in 2003, up from 36% in 2000 (*P* = .03). Among those screened, 76% (4913/6466) were up-to-date with screening, up from 68% (4146/6088) in 2000 (*P* = .01). 

Among the test categories analyzed for the ever screened group, colonoscopy (with or without FOBT) is the only test that increased in use with rates increasing from 38% in 2000 to 53% in 2003 (*P* = .02). Other tests showed a decrease in use, although the trend was not statistically significant.

In up-to-date screening behavior, trends were not statistically significant except for a decrease in sigmoidoscopy use (*P* = .01). There was increased use of colonoscopy screening between 2000 and 2003 (*P* = .05) and decreased use of FOBT (*P* = .08) and the combination of FOBT and sigmoidoscopy (*P* = .13).

### Predictors of screening use in 2003

Bivariate associations between individual characteristics and CRC screening use are presented in Table 2. Because 2003 and 2000 results were similar, we only describe 2003 results here. Individuals were more likely to be ever screened by any CRC test if they had a higher level of household income (*P* = .04), had a dental visit in the last year (*P* = .01), and had a usual source of care (*P* = .04). Individuals were more likely to be up-to-date with screening if they were white (*P* = .04), had a dental visit in the last year (*P* < .001), and had a usual source of care (*P* = .01). 

Results of the multivariate regressions (Tables Table 3 and Table 4) were similar to the bivariate associations seen in Table 2. Obesity (measured by body mass index) and self-reported health status were excluded from the regression model because they were not significantly associated with CRC screening use.

In 2003, individuals were more likely to be ever screened (Table 3) if they were older (odds ratio [OR], 1.04; 95% CI, 1.03–1.05; *P* < .001), had higher household income (OR, 1.06; 95% CI, 1.05–1.08; *P* < .001), had higher education levels (OR, 1.06; 95% CI, 1.05–1.08; *P* < .001), had insurance coverage (OR, 1.61; 95% CI, 1.27–2.05; *P* < .001 for private insurance; OR, 1.41; 95% CI, 1.06–1.87; *P* = .02 for Medicare; OR, 1.99; 95% CI, 1.44–2.50; *P* < .001 for Medicaid or other public insurance), had a usual source of care (OR, 2.06; 95% CI, 1.63–2.61; *P* < .001), had a dental visit in the past year (OR, 1.43; 95% CI, 1.28–1.61; *P* < .001), and had more than one chronic condition (OR, 1.37; 95% CI, 1.31–1.44; *P* < .001). Individuals were less likely to be screened if their race and ethnicity was Asian or other rather than white (OR, 0.44; 95% CI, 0.29–0.65; *P* < .001) and if they had more than one behavioral risk factor (OR, 0.81; 95% CI, 0.75–0.87; *P* < .001).

In 2003, individuals were more likely to be up-to-date (Table 4) with screening if they had higher household income (OR, 1.03; 95% CI, 1.01–1.06; *P =* .01), higher education (OR, 1.04; 95% CI, 1.00–1.07; *P* = .007), insurance coverage (OR, 1.68; 95% CI, 1.13–2.50; *P* = .01 for private insurance; OR, 2.07; 95% CI, 1.34–3.19; *P* < .001 for Medicare; OR, 2.18; 95% CI, 1.42–3.35; *P* < .001 for Medicaid or other public insurance), a usual source of care (OR, 2.59; 95% CI, 1.70–3.94; *P* < .001), and a dental visit in the past year (OR, 1.47; 95% CI, 1.21–1.78; *P* < .001).

### Predictors between 2000 and 2003

We examined whether there was a change in predictors by comparing the coefficients of the 2000 and 2003 models. Most predictors showed little effect on CRC screening use and were not statistically different between 2000 and 2003. In the ever screened group, there were two factors with a stronger effect in 2003 than in 2000, and these were having higher income (*P = *.003 in 2000 and *P* < .001 in 2003) and having Medicaid or other public insurance (*P* = .02 in 2000 and *P* <.001 in 2003) (Table 3). In the up-to-date screening group, factors with a stronger effect in 2003 than in 2000 were income level (*P *= .10 in 2000 and *P =* .01 in 2003), education level (*P* = .02 in 2000 and *P* = .007 in 2003), and having insurance (private insurance *P =* .07 in 2000 and *P* = .01 in 2003; Medicare *P =* .05 in 2000 and *P* < .001 in 2003; and Medicaid and other public insurance *P =* .66 in 2000 and *P* < .001 in 2003) (Table 4).

Predictors that remained the same for 2000 and 2003 for the ever screened group were age, having a usual source of care, having a dental visit in past year, having more than one behavioral risk factor, and having more than one chronic condition. Predictors that remained the same for the up-to-date screening group were having a usual source of care and having a dental visit in the previous year.

## Discussion

This study examined the rates and predictors of ever screened and up-to-date screening groups by using the most recent available national data, the 2000 and 2003 NHIS (15,16). In addition, we assessed whether predictors changed over time. We found that rates of ever screened and up-to-date screening increased by a modest amount between 2000 and 2003. Less than half the study population was up-to-date with screening (36% in 2000 and 42% in 2003). However, of the people who were ever screened, most are up-to-date on screening (68% in 2000 and 76% in 2003). We found that colonoscopy was the test used most frequently in both years. For both the ever screened and up-to-date groups we found an increase in rates of colonoscopy screening (*P* = .02 for the ever screened group and *P* = .05 for the up-to-date group) but a decrease in rates of use for FOBT (*P* = .08) and sigmoidoscopy (*P* = .01) for the up-to-date group.

Our findings about factors associated with up-to-date screening were consistent with existing literature. In 2003, the most influential socioeconomic health status predictors were 1) a usual source of care and insurance coverage, 2) a dental visit in the last year, 3) higher education, and 4) higher income. We found that among the insured there was little difference in the rates of CRC screening among privately insured, Medicare enrollees, and Medicaid or other public insurance enrollees.

We speculated that some predictors of CRC screening use may change over time because of changes in screening coverage and policies. For example, the effect of private insurance coverage might be stronger in 2003 than it was in 2000 because more states had laws requiring private health insurers to cover CRC screening (from seven states in 2000 to 18 states in 2003) (13). We also anticipated that the effect of Medicare coverage might be stronger in 2003 than in 2000 because of the 2000 enactment of Medicare coverage for colonoscopy screening. However, we did not find that having private insurance or Medicare coverage that covers CRC screening created statistically different results for 2003 compared with 2000. On the other hand, we found that positive effects of Medicaid and other public insurance coverage on CRC screening use was stronger in 2003 than in 2000. Medicaid coverage for CRC screening varies by state and is not standardized; further assessments are required to draw a conclusion about whether the observed differences in Medicaid coverage between the 2 years are due to changes in Medicaid coverage or a spill-over effect from the private and Medicare sectors to promote Medicaid coverage for CRC screening.

This study demonstrates how CRC screening use can be measured, a challenge faced by all CRC screening studies. There have been efforts to develop standardized self-reporting measures for CRC screening behavior to improve the quality of survey data (24). Because of the variety of CRC tests and screening time frames, there is a need to determine CRC test categories for individual tests and for combinations of tests. NHIS offers several advantages for studying CRC screening use because it provides complete information on which tests are used. For example, NHIS respondents were asked to identify the type of test used during their last colorectal examination (e.g., sigmoidoscopy, colonoscopy). Other national surveys, including the Medical Expenditure Panel Survey (MEPS) and Behavioral Risk Factor Surveillance System (BRFSS), do not differentiate between sigmoidoscopy and colonoscopy. Therefore, it is not feasible to study the use of colonoscopy, which is a much less well-studied test than FOBT and sigmoidoscopy. In addition, reports using MEPS or BRFSS are more likely to obtain higher estimates when a 10-year time frame is used to assess adherence for sigmoidoscopy and colonoscopy. Our rates of use and adherence were somewhat lower than those in BRFSS (25).

Future research should continue the search for other important predictors of CRC screening use. The current model could be extended further to examine health plan factors, contextual factors, and policy impact. Prior research found that individual health plan characteristics may influence the use of breast and cervical cancer screening services (26-28). Less is known about whether and how health plan factors influence CRC screening use. There is also a growing recognition that contextual factors (e.g., primary care physicians' beliefs and recommendations about CRC screening in the area where an individual lives, capacity for endoscopic CRC screening in the area of residence, the prevalence of managed care in an area where an individual lives) may affect the use of health services (18,29-32), but little is known about the extent to which CRC screening use is influenced by contextual factors. Finally, more research is needed to understand how policies, such as expanding Medicare coverage for colonoscopy screening, affect use of specific CRC tests over time.

This study has several limitations: 1) self-reported data may be inaccurate, although a prior study found good agreement between self-reported data and medical records for sigmoidoscopy and colonoscopy (33); 2) because it was not feasible for us to identify colonoscopies performed for diagnostic purposes (e.g., prompted by symptoms, done as a follow-up to other abnormal tests results) from those done only for screening, our estimates are higher than those in studies that examine CRC screening tests only (respondents may also inaccurately identify reasons for testing [33]); 3) we were unable to examine other key predictors identified in the literature (e.g., test preferences of patients and physicians, physician recommendations, supply-side factors such as capacity of local health care facilities for CRC screening) because NHIS lacks such data. (Information on physicians’ recommendations was collected only in the 2000 NHIS and only for respondents who were never screened or who were not screened within the recommended time frame.)

Rates of ever screened and up-to-date screening have increased between 2000 and 2003 but only modestly. Although screening rates remain low, most people who get screened at all are up-to-date with screening. We found that predictors of screening were stable over time despite changes in CRC screening policies. The most influential socioeconomic predictors are having insurance coverage, a higher income, a usual source of care, and a dental visit in the past year. Further research is needed to uncover barriers to CRC screening and to develop strategies to overcome these barriers.

## Figures and Tables

**Table 1:** Unadjusted^a^ Rates of Colorectal Cancer Screening Among Adults Aged 50 Years and Older in the 2000 (N =11,574) and 2003 (N =11,779) National Health Interview Surveys

Categories	**No. (%)**		***P* b**

	**2000**	**2003**	
Unscreened	5486 (47)	5313 (45)	.10
Ever screened^c^ ^d^	6088 (53)	6466 (55)	.10
FOBT only	1752 (29)	1597 (25)	.14
Sigmoidoscopy only	469 (8)	358 (6)	.16
Colonoscopy (with or without FOBT)	2289 (38)	3428 (53)	.02
FOBT and sigmoidoscopy	747 (12)	567 (9)	.15
Do not know or other test	831 (14)	516 (8)	.11
Not up-to-date	1942 (32)	1553 (24)	.01
Up-to-date	4146 (68)	4913 (76)	.01
FOBT only	1071 (26)	782 (16)	.08
Sigmoidoscopy only	631 (15)	496 (10)	.01
Colonoscopy (with or without FOBT)	2118 (51)	3427 (70)	.05
FOBT and sigmoidoscopy	326 (8)	208 (4)	.13
Up-to-date (total samples)^e^	4146 (36)	4913 (42)	.03

FOBT indicates fecal occult blood test.

a Data were not controlled for other confounders.

b To account for the survey design, an F statistic based on Rao and Scott correction was used instead of the χ^2^ test.

c Ever screened is defined as having ever been screened using FOBT, sigmoidoscopy, or colonoscopy.

d Test categories are mutually exclusive.

e Up-to-date screening was defined as having an FOBT in the past 12 months, a sigmoidoscopy in the past 5 years, or a colonoscopy in the past 10 years.

**Table 2:** Weighted^a^ Bivariate Associations Between Individual Characteristics and Colorectal Cancer Screening Rates Among Adults Aged 50 Years and Older in the 2000 and 2003 National Health Interview Surveys (NHIS)

**Variables**	**Ever Screened Among Total Sample**				**Up-to-Date Screening Among Ever Screened**			

	**2000, % (N = 11,574)**	***P* b**	**2003, % (N = 11,779)**	***P***	**2000, % (n = 6088)**	***P***	**2003, % (n = 6466)**	***P***
**Age, y**								
50-64	49	.08	51	.09	67	.21	77	.48
65-74	63		66		73		78	
≥75	59		63		68		75	
**Sex**								
Male	54	.64	57	.47	68	.07	79	.20
Female	54		56		69		75	
**Race and ethnicity**								
Hispanic	36	.09	39	.13	68	.10	77	.36
White	57		59		69		77	
African American	47		51		67		75	
Other	41		40		59		71	
**Race**								
White	57	.04	59	.10	69	.09	77	.04
Nonwhite	42		45		66		75	
**Household income (% federal poverty level)**								
Low (<200)	48	.07	48	.04	66	.38	72	.17
Medium (200-399)	55		59		68		75	
High (≥400)	56		58		70		79	
**Education**								
<High school graduate	44	.06	45	.06	64	.20	71	.08
High school graduate	53		55		68		76	
≥College graduate	60		62		71		79	
**Dental visit in last year**								
Yes	60	.04	61	.01	71	.13	80	<.001
No	44		48		64		71	
**Insurance coverage**								
Private	57	.04	60	.14	70	.22	78	.07
Medicare	54		57		70		77	
Medicaid or other public insurance	45		51		60		76	
Uninsured	26		28		58		59	
**Usual source of care**								
Yes	56	.02	58	.04	69	.14	78	.01
No	26		25		51		50	
**Number of behavioral risk factors^c^**								
0	63	.08	65	.12	73	.28	78	.25
1	53		55		68		77	
2	47		47		64		74	
3	42		52		64		83	
**Body mass index^d^**								
Underweight or normal	52	.15	55	.53	69	.75	76	.66
Overweight or obese	55		57		69		77	
**Number of chronic conditions^e^**								
0	44	.02	44	.08	66	.18	76	.46
1	53		57		71		76	
2	62		62		68		79	
≥3	67		69		69		76	
**Self-reported health status**								
Very good	53	.39	56	.41	70	.50	78	.52
Good	54		57		69		77	
Fair	55		54		67		74	
Poor	56		56		67		75	

a Data were weighted to reflect U.S. civilian, noninstitutionalized population but not for other confounders.

b To account for the survey design, an F statistic based on Rao and Scott correction was used instead of the χ^2^ test.

c Behavioral risk factors include 1) alcohol consumption >720 drinks per year for men and >360 drinks for women, 2) smoking, and 3) lack of physical activity.

d Underweight or normal sample includes individuals with a body mass index of less than 25 kg/m^2^; overweight or obese sample includes individuals with a body mass index of 25 kg/m^2^ or more.

e Chronic conditions include diabetes, arthritis, ulcers, cardiovascular disease, hypertension, and asthma or emphysema.

**Table 3:** Multivariate Logistic Regression Analysis of Adjusted^a^ Rates of Adults Aged 50 Years and Older Who Were Ever Screened for Colorectal Cancer in the 2000 and 2003 National Health Interview Surveys (NHIS)

**Correlates**	**Adults Ever Screened for Colorectal Cancer^a^**				

	**2000 (n = 8377)**		**2003 (n = 8271)**		**β2000:2003**

	**OR (95% CI)**	***P***	**OR (95% CI)**	***P***	**Wald F test_1,339_ (*P*)^b^**
Age^c^	1.03 (1.02-1.03)	<.001	1.04 (1.03-1.05)	<.001	3.93 (.05)
Sex					
Female	1.04 (0.93-1.16)	.50	0.95 (0.85-1.07)	.41	.94 (.33)
Male	Ref	—	Ref	—	—
Race and ethnicity					
Hispanic	0.67 (0.55-0.80)	<.001	0.84 (0.70-1.02)	.08	2.84 (.09)
White	Ref	—	Ref	—	—­
African American	0.88 (0.74-1.05)	.16	0.98 (0.82-1.17)	.80	.74 (.39)
Asian or other	0.68 (0.48-0.96)	.03	0.44 (0.29-0.65)	<.001	2.61 (.11)
Household income^d^	1.03 (1.00-1.05)	.003	1.06 (1.05-1.08)	<.001	8.69 (.003)
Education level^c^	1.06 (1.04-1.08)	<.001	1.06 (1.05-1.08)	<.001	.02 (.88)
Insurance coverage					
Private insurance	1.76 (1.38-2.24)	<.001	1.61 (1.27-2.05)	<.001	.13 (.72)
Medicare	1.54 (1.15-2.05)	.003	1.41 (1.06-1.87)	.02	.09 (.76)
Medicaid or other public insurance	1.37 (1.04-1.81)	.02	1.99 (1.44-2.50)	<.001	4.24 (.04)
Uninsured	Ref	—	Ref	—	—
Having usual source of care					
Yes	2.21 (1.76-2.76)	<.001	2.06 (1.63-2.61)	<.001	.27 (.60)
No	Ref	—	Ref	—	—
Dental visit in last year					
Yes	1.66 (1.47-1.87)	<.001	1.43 (1.28-1.61)	<.001	3.51 (.06)
No	Ref	—	Ref	—	—
Number of behavioral risk factors^e^	0.86 (0.80-0.93)	<.001	0.81 (0.75-0.87)	<.001	1.10 (.30)
Number of chronic conditions^f^	1.36 (1.30-1.43)	<.001	1.37 (1.31-1.44)	<.001	.01 (.94)

OR indicates odds ratio; CI, confidence interval; Ref, reference group.

a Data were weighted to reflect the U.S. civilian, noninstutionalized population for each year and SE adjustment to account for the complex survey design.

b Significant *P* values (<.05) reject the null hypothesis that the coefficient in the 2000 model is equal to the coefficient in the 2003 model.

c Age and education were treated as continuous variables and were measured in years (age range, 50 to 85 years; mean 64, SE = 10.62).

d Poverty level was treated as an ordinal variable and ranged from 1 to 14, with 1 = under 50% poverty level and 14 = 500% or greater.

e Behavioral risk factors include 1) alcohol consumption >720 drinks per year for men and >360 drinks for women, 2) smoking, and 3) lack of physical activity.

f Chronic conditions include diabetes, arthritis, ulcers, cardiovascular disease, hypertension, and asthma or emphysema.

**Table 4:** Multivariate Logistic Regression Analysis of Adjusted^a^ Rates of Adults Aged 50 Years and Older Who Are Up-to-Date for Colorectal Cancer Screening in the 2000 and 2003 National Health Interview Surveys (NHIS)^a^

**Correlates**	**Adults Up-to-Date for Colorectal Cancer Screening**				

	**2000 (n = 4459)**		**2003 (n = 4694)**		**β2000:2003**

	**OR (95% CI)**	***P***	**OR (95% CI)**	***P***	**Wald F test_1,339_ (*P*)^b^**
Age^c^	1.00 (1.00-1.01)	.22	1.00 (1.00-1.01)	.34	.12 (.73)
Sex					
Female	0.94 (0.84-1.10)	.46	0.89 (0.76-1.05)	.16	.13 (.72)
Male	Ref	—	Ref	—	—
Race and ethnicity					
Hispanic	1.20 (0.91-1.59)	.20	1.26 (0.93-1.69)	.14	.00^d^ (.99)
White	Ref	—	Ref	—	—
African American	1.00 (0.78-1.28)	.97	1.05 (0.80-1.37)	.72	.04 (.84)
Asian or other	0.65 (0.39-1.09)	.10	0.88 (0.52-1.48)	.63	.80 (.37)
Household income^e^	1.00 (0.98-1.03)	.10	1.03 (1.01-1.06)	.01	2.24 (.14)
Education level^c^	1.03 (1.00-1.06)	.02	1.04 (1.00-1.07)	.007	.00^f^ (.98)
Insurance coverage					
Private insurance	1.39 (0.98-1.99)	.07	1.68 (1.13-2.50)	.01	.45 (.50)
Medicare	1.50 (1.01-2.23)	.05	2.07 (1.34-3.19)	<.001	.82 (.37)
Medicaid or other public insurance	1.10 (0.72-1.68)	.66	2.18 (1.42-3.35)	<.001	4.14 (.04)
Uninsured	Ref	—	Ref	—	—
Having usual source of care					
Yes	2.05 (1.43-2.95)	<.001	2.59 (1.70-3.94)	<.001	.25 (.62)
No	Ref	—	Ref	—	—
Dental visit in last year					
Yes	1.32 (1.12-1.57)	.001	1.47 (1.21-1.78)	<.001	.13 (.72)
No	Ref	—	Ref	—	—
Number of behavioral risk factors^g^	0.84 (0.75-0.94)	.003	0.99 (0.87-1.12)	.84	4.31 (.04 )
Number of chronic conditions^h^	1.02 (0.96-1.08)	.56	1.06 (0.99-1.14)	.19	.44 (.51 )

OR indicates odds ratio; CI, confidence interval; Ref, reference group.

a Data were weighted to reflect the U.S. civilian, noninstutionalized population for each year and SE adjustment to account for the complex survey design.

b Significant *P* values (<.05) reject the null hypothesis that the coefficient in the 2000 model is equal to the coefficient in the 2003 model.

c Age and education were treated as continuous variables and were measured in years (age range, 50 to 85 years; mean, 64, SE = 10.62). Education F test value = .000507.

d Hispanic F test value = .000075.

e Poverty level was treated as an ordinal variable and ranged from 1 to 14, with 1 = under 50% federal poverty level and 14 = 500% or greater.

f Education F test value = .000507.

g Behavioral risk factors include 1) alcohol consumption >720 drinks per year for men and >360 drinks for women, 2) smoking, and 3) lack of physical activity.

h Chronic conditions include diabetes, arthritis, ulcers, cardiovascular disease, hypertension, and asthma or emphysema.
